# P2Y_12_ inhibition, adenosine signaling, and bone remodeling: differential skeletal pharmacology of antiplatelet agents

**DOI:** 10.3389/fphar.2026.1897702

**Published:** 2026-10-12

**Authors:** Zhuoqun Ma, Wei Luo, Yuanbo Li, Hong Li, Chaomin Li

**Affiliations:** Second Ward, Cardiovascular Disease Treatment Center, Honghui Hospital, Xi’an Jiaotong University, Xi’an, China

**Keywords:** antiplatelet therapy, bone metabolism, fracture healing, P2Y_12_ receptor, ticagrelor

## Abstract

Cardiovascular disease, osteoporosis, and fragility fractures frequently coexist in older adults, creating an important overlap between antiplatelet therapy and orthopedic care. This narrative review examines mechanistic, preclinical, observational, and perioperative evidence concerning the skeletal effects of aspirin, P2Y_12_ inhibitors, and other clinically relevant antiplatelet agents. Purinergic signaling provides biological plausibility for interactions between platelet inhibition and bone remodeling because P2Y_12_, P2Y_1_, P2X_7_, adenosine receptors, and equilibrative nucleoside transporter 1 (ENT1) regulate osteoblast differentiation, osteoclast activity, inflammatory responses, and tissue repair. Aspirin shows dose- and context-dependent skeletal effects, whereas clopidogrel has been associated with impaired osteoblast mineralization in preclinical studies and conflicting fracture-risk estimates in observational cohorts. Direct skeletal evidence for prasugrel and other agents remains limited. Selected preclinical studies suggest that ticagrelor-associated ENT1 inhibition can enhance adenosine-dependent osteogenic signaling; however, clinical evidence is limited to one retrospective cohort in which ticagrelor-based dual antiplatelet therapy (DAPT) was associated with lower fracture risk than clopidogrel-based DAPT, while prospective skeletal outcome data are lacking. Perioperative antiplatelet management in orthopedic surgery should remain guided by cardiovascular indications, thrombotic risk, and bleeding risk. A conceptual framework may help define priorities for evaluating skeletal outcomes during antiplatelet therapy. However, current evidence is insufficient to support antiplatelet selection or switching on the basis of presumed skeletal effects.

## 1 Introduction

### 1.1 Cardiovascular disease and osteoporotic fractures in aging

Global population aging has created a dual burden of cardiovascular disease (CVD), osteoporosis, and fragility fractures in older adults (Tu et al., 2026). Epidemiological studies consistently show that low bone mineral density (BMD), osteoporosis, and vertebral or hip fractures are associated with increased cardiovascular risk (Veronese et al., 2017). Population-based data from Taiwan associated low BMD with increased all-cause, cardiovascular, and cancer mortality (Cai et al., 2024), whereas observational and Mendelian-randomization analyses found no robust evidence that lower BMD causally increases coronary artery calcification (Lu H. et al., 2024). Importantly, fragility fractures are followed by a marked rise in major adverse cardiovascular events (MACE). A UK population study found substantially higher MACE incidence within 1 year after fragility fracture than after newly diagnosed osteoporosis alone (Pineda-Moncusí et al., 2022), and a Hong Kong cohort further showed that hip fracture increased one-year MACE risk, especially within the first 90 days (Hsu et al., 2022).

This association is bidirectional. A meta-analysis of longitudinal studies demonstrated that low BMD and baseline fractures predicted subsequent CVD events (Veronese et al., 2017), while large prospective cohorts associated low BMD with cardiovascular mortality and prevalent vertebral fracture with incident cardiovascular events (Cai et al., 2024; Schousboe et al., 2025). Conversely, cardiovascular events may also increase later fracture risk, as shown by an Olmsted County study reporting a strengthening association between myocardial infarction and subsequent osteoporotic fracture over recent decades (Gerber et al., 2011). Hip fracture patients are particularly vulnerable to acute cardiovascular complications, with Danish nationwide data showing markedly increased short-term risks of myocardial infarction and stroke after hip fracture (Pedersen et al., 2017). Together, these findings indicate that cardiovascular disease and skeletal fragility frequently coexist and may reinforce each other in aging populations.

### 1.2 Antiplatelet therapy in orthopedic patients

The convergence of cardiovascular disease and osteoporotic fractures means that many elderly fracture patients are already receiving antiplatelet therapy for coronary artery disease or post-percutaneous coronary intervention management. Population-based data show that antithrombotic use among hip fracture patients has increased substantially over time, with antiplatelet agents representing a major subgroup (Madsen et al., 2016; Mahmood et al., 2021; Vleeshouwers et al., 2025). Preoperative antiplatelet use has been associated with higher transfusion rates and short-term mortality in large cohorts, although adjusted analyses suggest that these outcomes may largely reflect underlying cardiovascular comorbidity rather than a direct drug effect (Daugaard et al., 2019; Mahmood et al., 2021). Recent meta-analyses indicate that aspirin or broader antiplatelet exposure does not clearly increase perioperative mortality, bleeding events, or hematoma formation in hip or femoral fracture surgery (Xue et al., 2025). The main clinical dilemma is whether antiplatelet therapy should delay urgent fracture surgery. Evidence from observational meta-analyses suggests that early surgery in patients receiving aspirin, clopidogrel, or dual antiplatelet therapy may modestly increase blood loss but reduces complications, length of stay, and possibly mortality compared with delayed surgery (Lu W. et al., 2024; Tang et al., 2025).

### 1.3 The neglected skeletal effects of antiplatelet drugs

Current antiplatelet prescribing is mainly guided by cardiovascular efficacy and bleeding risk, while potential skeletal effects are rarely considered (Rao et al., 2025). This gap is biologically relevant because the P2Y_12_ receptor is expressed not only in platelets but also in osteoblasts and osteoclasts, and P2ry12 deletion in mice reduces osteoclast resorptive activity and protects against several forms of bone loss (Su et al., 2012; Syberg et al., 2012). Preclinical evidence suggests that individual P2Y_12_ inhibitors may have divergent skeletal effects (Mediero et al., 2016). Clopidogrel inhibits osteoblast mineralization and reduces trabecular bone mass in animal models (Syberg et al., 2012), although epidemiological findings on fracture risk remain conflicting and may be confounded by cardiovascular comorbidity (Jørgensen et al., 2012; Vestergaard et al., 2012). By contrast, ticagrelor inhibits equilibrative nucleoside transporter 1 (ENT1), increases extracellular adenosine, and promotes A2A receptor-dependent osteogenesis and bone regeneration in preclinical models (Mediero et al., 2016). Aspirin also shows mixed skeletal effects, with experimental evidence suggesting both bone-forming and delayed-healing effects, whereas randomized clinical data show no clear fracture benefit (de Souza Rendohl et al., 2021; Barker et al., 2022).

### 1.4 Scope and objectives

This narrative review evaluates the potential effects of antiplatelet agents on bone metabolism, fracture healing, and orthopedic perioperative care, with particular emphasis on purinergic signaling as a mechanistic bridge between platelet inhibition and skeletal biology. It also examines the translational limitations of the available evidence and identifies the knowledge gaps that must be addressed before skeletal considerations can inform antiplatelet management.

Accordingly, the review is organized around six interrelated themes: (1) purinergic and adenosine signaling in platelet function and skeletal remodeling; (2) the drug-specific skeletal profiles of aspirin, clopidogrel, ticagrelor, and prasugrel, with brief consideration of cangrelor, cilostazol, and dipyridamole; (3) preclinical and clinical findings on bone metabolism, fracture healing, bone regeneration, and osseointegration; (4) perioperative antiplatelet management in orthopedic surgery; (5) clinical contexts in which thrombotic, bleeding, and skeletal risks intersect; and (6) translational limitations and research priorities that must be addressed before skeletal outcomes can inform clinical practice.

### 1.5 Literature search and study selection

A focused literature search was conducted in PubMed/MEDLINE, Scopus, and the Web of Science Core Collection from database inception through August 2026. The main search combined antiplatelet terms (aspirin OR clopidogrel OR ticagrelor OR prasugrel) with skeletal terms (bone OR osteoblast OR osteoclast OR “bone remodeling” OR osteoporosis OR “bone mineral density” OR “fracture risk” OR “fracture healing” OR “bone regeneration” OR “implant integration”), using AND between the two groups. Supplementary targeted searches addressed cangrelor, cilostazol, dipyridamole, purinergic signaling, P2Y_12_, adenosine, ENT1, and perioperative antiplatelet management. Reference lists of relevant articles and reviews were also screened.

English-language experimental studies, clinical trials, observational studies, systematic reviews, meta-analyses, and clinical guidelines relevant to skeletal biology or orthopedic antiplatelet management were considered. Non-English publications, duplicate reports, conference abstracts without sufficient data, and studies without relevant skeletal, mechanistic, or perioperative outcomes were excluded. Study selection was performed by two authors, with title and abstract screening followed by full-text assessment of potentially relevant articles. Given the heterogeneity of study designs and outcomes, evidence was synthesized narratively by drug, evidence level, and skeletal endpoint; no formal risk-of-bias assessment or meta-analysis was performed.

## 2 Purinergic signaling at the crossroads of hemostasis and bone homeostasis

### 2.1 Purinergic receptor families in platelets and bone cells

Purinergic receptors comprise two major families, P2Y G protein-coupled receptors and P2X ligand-gated ion channels, which are activated by extracellular ATP and ADP released during tissue injury or mechanical loading (Kringelbach et al., 2015). In platelets, P2Y_12_ is the major ADP receptor driving platelet aggregation and serves as the therapeutic target of antiplatelet agents such as clopidogrel and ticagrelor, while P2Y_1_ and P2X_1_ regulate platelet activation and calcium influx (Kamran et al., 2021). In bone, multiple P2Y and P2X receptor subtypes are expressed in osteoblasts, osteoclasts, and osteocytes, highlighting their broad role in skeletal remodeling (Agrawal et al., 2017; Mikolajewicz et al., 2021). P2Y_2_ is a key mechanosensor that regulates osteoblast differentiation and ATP-induced ERK1/2 signaling, whereas P2Y_6_ and P2Y_12_ promote osteoclast formation and bone resorption (Mikolajewicz et al., 2021). Among P2X receptors, P2X_7_ is central to skeletal mechanotransduction, mediating ATP-dependent calcium signaling, prostaglandin E2 release, and adaptive bone remodeling in response to mechanical stress (Agrawal et al., 2017). As summarized in Figure 1, extracellular nucleotide release, adenosine generation, and receptor-specific signaling provide a mechanistically plausible interface between platelet inhibition and skeletal remodeling, although their clinical skeletal relevance remains uncertain.

**FIGURE 1:**
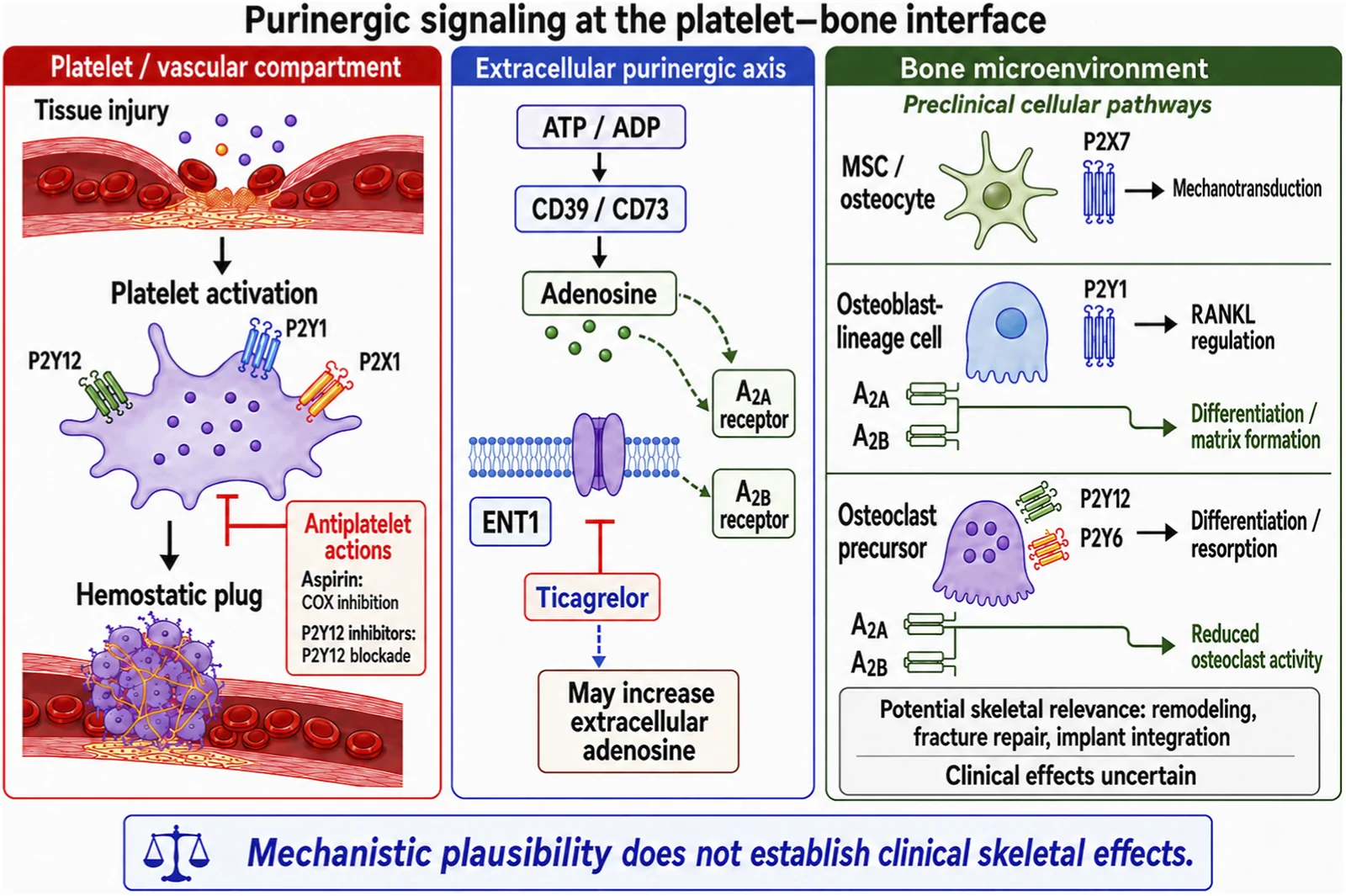
Purinergic signaling at the platelet–bone interface. Tissue injury activates platelets and promotes extracellular ATP and ADP release. P2Y_12_, P2Y_1_, and P2X_1_ contribute to platelet activation, whereas aspirin and P2Y_12_ inhibitors suppress platelet-dependent signaling through distinct mechanisms. CD39 and CD73 sequentially convert extracellular nucleotides to adenosine, while ENT1 regulates adenosine uptake. Ticagrelor inhibits ENT1 and may increase extracellular adenosine under selected experimental conditions (Cattaneo et al., 2014; Aungraheeta et al., 2016). In preclinical models, purinergic and adenosine receptor signaling has been associated with mechanotransduction, osteoblast-lineage responses, osteoclast differentiation, and bone resorption (Agrawal and Jørgensen, 2021; Mediero et al., 2016). Solid arrows indicate directly supported biochemical or cellular relationships, dashed arrows indicate proposed or context-dependent links whose skeletal consequences remain preclinical, and red T-bars indicate inhibition. These mechanisms establish biological plausibility but do not demonstrate clinical effects on bone remodeling, fracture repair, or implant integration.

### 2.2 P2Y_12_ receptor signaling in platelets and bone remodeling

The P2Y_12_ receptor is a Gi-coupled ADP receptor that amplifies platelet activation and stabilizes thrombus formation. In platelets, P2Y_12_ signaling inhibits adenylyl cyclase, reduces intracellular cAMP, activates RAP1, and promotes integrin αIIbβ3 activation and platelet aggregation (Kamran et al., 2021). This receptor is the pharmacological target of clopidogrel, prasugrel, ticagrelor, and cangrelor (Kamran et al., 2021).

Beyond hemostasis, P2Y_12_ is expressed in osteoclast-lineage cells and contributes to ADP-dependent osteoclast adhesion and resorptive activity (Su et al., 2012; Lenertz et al., 2015). P2ry12-deficient mice exhibit reduced osteoclast function and protection against several forms of pathological bone loss, while pharmacological P2Y_12_ blockade attenuates osteolysis in experimental models (Su et al., 2012). In mesenchymal stromal cells from postmenopausal women, combined silencing of P2Y_12_ and P2Y_13_ restored ADP-induced P2Y_1_-mediated osteogenic commitment (Bessa-Andrês et al., 2025). However, these experimental findings do not establish the skeletal effects of clinically prescribed P2Y_12_ inhibitors.

### 2.3 P2Y_1_ receptor and osteoblast-mediated RANKL expression

The P2Y_1_ receptor, expressed on osteoblasts and osteoclasts, plays a central role in linking extracellular nucleotide signaling to bone resorption (McCutcheon et al., 2020). Activation of P2Y_1_ on osteoblasts enhances receptor activator of nuclear factor-κB ligand (RANKL) expression, indirectly promoting osteoclast formation and activity (McCutcheon et al., 2020). Extracellular ADP, a major ATP degradation product released during tissue injury or mechanical stress, stimulates osteoclast-mediated resorption at nanomolar to micromolar concentrations, an effect blocked by the selective P2Y_1_ antagonist MRS 2179 (Hoebertz et al., 2001; Agrawal and Jørgensen, 2021). In human periodontal ligament cells, ATP triggers RANKL expression through a P2Y_1_–cyclooxygenase-dependent pathway, demonstrating that P2Y_1_ couples mechanical or inflammatory nucleotide release to osteoclastogenic signaling (Kyawsoewin et al., 2024). Co-activation of P2Y_1_ and parathyroid hormone receptors synergistically enhances osteoblastic gene expression, integrating local purinergic cues with systemic hormonal regulation (Xing et al., 2021). Apoptotic osteocytes release ATP via pannexin-1 channels, activating P2Y_1_ and inducing RANKL in neighboring osteocytes, initiating targeted remodeling at microdamage sites (McCutcheon et al., 2020). Blocking P2Y_1_ signaling reduces osteoclast formation and bone resorption *in vitro*, suggesting potential therapeutic applications to limit pathologic bone loss in inflammatory or metabolic bone disorders (Bessa-Andrês et al., 2025).

### 2.4 Adenosine receptors in osteoblast and osteoclast regulation

Adenosine, generated extracellularly during tissue injury, hypoxia, and mechanical stress, regulates bone remodeling primarily through the A2A and A2B adenosine receptors (A2AR and A2BR) (Borhani et al., 2019). A2AR activation in osteoblasts promotes Akt phosphorylation and β-catenin nuclear translocation, thereby enhancing osteoblast differentiation and bone formation (Mediero et al., 2016). A2AR signaling also modulates semaphorin-mediated osteoclast–osteoblast communication and promotes bone regeneration in murine calvarial defect models, with osteogenic effects comparable to BMP-2 (Mediero et al., 2018; Zheng and Wang, 2020). Consistently, A2AR-deficient mice exhibit reduced bone mass and impaired fracture healing (Strazzulla and Cronstein, 2016). A2BR exerts complementary effects by simultaneously stimulating osteoblast activity and suppressing osteoclastogenesis (Kim et al., 2017). Activation of A2BR inhibits RANKL-induced osteoclast differentiation through suppression of ERK1/2, p38 MAPK, and NF-κB signaling pathways, while also reducing osteoclast fusion and resorptive activity (Kim et al., 2017). In ovariectomized mice and multiple myeloma-associated bone disease models, pharmacologic A2BR agonism attenuates bone loss and promotes osteoblast differentiation (Shih et al., 2019). Genetic deletion of A2BR results in reduced bone mineral density and increased osteoclast activity, confirming its essential role in maintaining skeletal homeostasis (Corciulo et al., 2016).

### 2.5 ENT1 as a shared pharmacological target

ENT1 is the principal bidirectional transporter mediating cellular adenosine uptake in platelets, erythrocytes, and skeletal cells (Aungraheeta et al., 2016). Ticagrelor selectively inhibits ENT1, unlike clopidogrel, prasugrel, or cangrelor, thereby prolonging extracellular adenosine availability and enhancing adenosine receptor signaling in cardiovascular and inflammatory tissues (Cattaneo et al., 2014; Akkaif et al., 2021). Through increased activation of A2A and A2B receptors, this mechanism contributes to ticagrelor’s pleiotropic effects beyond P2Y_12_ inhibition (Cattaneo et al., 2014). ENT1 also plays an important role in skeletal homeostasis. ENT1-deficient mice develop age-dependent reductions in bone mineral density accompanied by increased osteoclast activity, while paradoxically exhibiting progressive ectopic spinal and ligamentous mineralization resembling diffuse idiopathic skeletal hyperostosis (DISH) (Daniels et al., 2015). These abnormalities are associated with elevated extracellular adenosine levels, impaired pyrophosphate regulation, and reduced expression of mineralization inhibitors such as ENPP1 and ANK (Ii et al., 2016). Furthermore, annulus fibrosus cells from ENT1-null mice show enhanced alkaline phosphatase activity and osteogenic mineralization (Ii et al., 2016). Collectively, these findings suggest that ticagrelor-mediated ENT1 inhibition may influence bone remodeling through adenosine-dependent pathways distinct from those targeted by other P2Y_12_ antagonists.

## 3 Drug-specific effects on bone metabolism: a pharmacological dissection

As summarized in Figure 2, the volume, directness, and clinical applicability of skeletal evidence vary substantially across antiplatelet agents, and the strength of preclinical evidence should not be interpreted as clinical skeletal efficacy.

**FIGURE 2:**
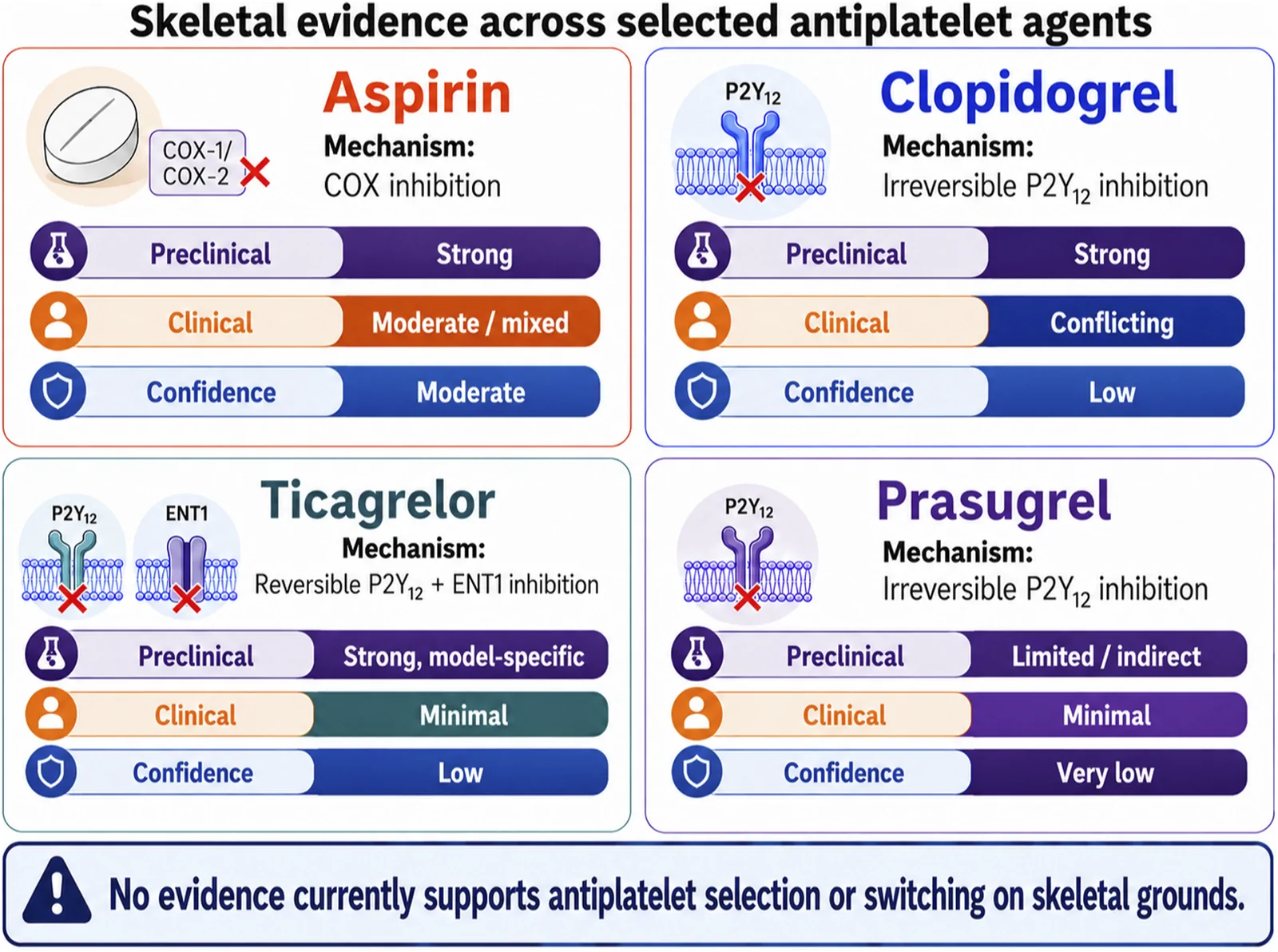
Skeletal evidence across selected antiplatelet agents. Evidence levels are shown separately for preclinical studies, clinical studies, and overall confidence. Aspirin findings vary by dose, route, and duration, and the ASPREE trial did not demonstrate a reduction in fracture risk (Lack et al., 2013; Barker et al., 2022). Clopidogrel has substantial but model-dependent experimental evidence, while clinical fracture associations remain conflicting (Jørgensen et al., 2012; 2017; Syberg et al., 2012). Ticagrelor evidence is largely model-specific, including *in vitro* experiments and local delivery in a murine calvarial-defect model; clinical evidence is limited to one retrospective cohort, with no prospective skeletal outcome studies (Mediero et al., 2016; Lui et al., 2024). Direct skeletal evidence for prasugrel remains limited. Ratings represent a structured qualitative assessment rather than a formal GRADE evaluation. No category supports antiplatelet selection or switching on skeletal grounds. Colors distinguish agents and evidence domains and do not indicate relative clinical efficacy or safety.

### 3.1 Aspirin: COX inhibition and bone metabolism

#### 3.1.1 Experimental evidence

Experimental effects of aspirin on bone are dependent on concentration, route, duration, and model (Xie et al., 2019). At low concentrations, aspirin enhanced osteogenic differentiation of human dental pulp stem cells, including alkaline phosphatase activity, mineralized nodule formation, and expression of osteogenic markers (Vukovic et al., 2022). In high-fat-fed ovariectomized rats, aspirin attenuated bone loss by increasing bone formation, although mechanical properties were not fully restored (Lin et al., 2016). *In vitro*, aspirin inhibited RANKL-induced osteoclastogenesis through suppression of NF-κB, MAPK, and NFATc1-related signaling (Zeng et al., 2016; Wu et al., 2019). These findings establish context-dependent biological activity but do not demonstrate a net skeletal benefit in humans.

#### 3.1.2 Fracture-risk evidence

Human evidence relating aspirin to fracture risk is conflicting. A meta-analysis of six observational studies reported 17% lower odds of any fracture among aspirin users, but heterogeneity was substantial and residual confounding could not be excluded (Barker et al., 2020). Conversely, a Danish population-based study associated acetylsalicylic acid use with increased fracture risk (Vestergaard et al., 2012). In the randomized ASPREE trial involving 16,703 older adults, daily aspirin at 100 mg did not reduce fracture risk over a median follow-up of 4.6 years (HR 0.97, 95% CI 0.87–1.06) (Barker et al., 2022). Current evidence therefore does not support aspirin use for fracture prevention.

#### 3.1.3 Bone mineral density evidence

Observational studies examining aspirin use and BMD have produced inconsistent findings. An NHANES analysis of adults aged 50–80 years associated low-dose aspirin use with modestly higher BMD at several femoral and lumbar sites (Liu et al., 2022). In contrast, a Dutch general-population study found no association between chronic low-dose aspirin use and vertebral or femoral BMD after multivariable adjustment, including among postmenopausal women (Bonten et al., 2017). An exploratory meta-analysis found only small, statistically nonsignificant differences in hip and lumbar-spine BMD between aspirin users and nonusers (Barker et al., 2020). Because these BMD findings are observational, causality cannot be established. Sources of heterogeneity across aspirin-related fracture and BMD studies, including dose, exposure duration, population characteristics, and outcome definitions, are discussed in Section 4.8.

### 3.2 Clopidogrel: irreversible P2Y_12_ antagonism and skeletal effects

#### 3.2.1 *In vitro* evidence

Direct *in vitro* evidence for clopidogrel remains limited and model dependent. In primary murine bone-cell cultures, clopidogrel at 10–25 μM concentration-dependently reduced osteoblast proliferation, alkaline phosphatase activity, collagen production, and mineralized nodule formation. Concentrations of ≥1 μM also inhibited osteoclast formation and resorptive activity, suggesting suppression of overall bone turnover (Syberg et al., 2012). A subsequent study using human adipose-derived mesenchymal stem cells directly compared clopidogrel and ticagrelor at 1.8 μM. Ticagrelor produced greater mineralization, alkaline phosphatase activity, and osteogenic-marker expression, whereas both agents reduced inflammatory markers (Mohamed et al., 2024). Differences in species, cell source, concentration, and experimental design limit direct comparison, and neither study establishes clinical skeletal superiority or the net effect of clopidogrel on human bone remodeling or fracture healing.

#### 3.2.2 *In vivo* evidence

*In vivo* findings for clopidogrel are also model dependent. In mice receiving clopidogrel for 4 weeks, bone mineral density and trabecular bone volume were reduced, whereas cortical bone was less affected (Syberg et al., 2012). In contrast, a periodontitis model associated clopidogrel exposure with increased mesenchymal stromal-cell and osteoblast numbers during the reparative phase, with little effect in the absence of prior inflammatory stimulation (Coimbra et al., 2015). The active metabolite of clopidogrel also increased osteoblast differentiation and local bone formation in a murine calvarial-defect model through an A2B receptor–dependent mechanism (Mediero et al., 2016). These divergent findings involve different exposures and skeletal models and cannot be directly extrapolated to systemic clopidogrel treatment during human long-bone healing.

#### 3.2.3 Epidemiological evidence

Epidemiological studies on clopidogrel and fracture risk remain inconsistent. A Danish nationwide cohort study of 77,503 clopidogrel users reported increased overall and osteoporotic fracture risk, particularly with treatment durations >1 year, and identified a biphasic dose-response relationship in which very low exposure was associated with lower fracture risk (Jørgensen et al., 2012). However, subsequent analyses restricted to stroke and TIA patients found no increased fracture risk after adjustment for confounders and even suggested a 10%–35% reduction in fracture risk among clopidogrel users (Jørgensen et al., 2017). Another Danish case-control study found no significant association between clopidogrel and fractures (Vestergaard et al., 2012). Cardiovascular comorbidity, stroke-related disability, treatment duration, and interindividual variability in clopidogrel metabolism may contribute to these conflicting findings, as discussed further in Section 4.8.

#### 3.2.4 The P2Y_12_ paradox

A discrepancy exists between genetic P2Y_12_ deficiency and pharmacological inhibition with clopidogrel. P2ry12-deficient mice exhibit reduced osteoclast activity and protection against age-related, inflammatory, tumor-associated, and ovariectomy-induced bone loss (Su et al., 2012). In contrast, clopidogrel treatment in wild-type mice reduced bone mineral density and trabecular bone volume, while *in vitro* exposure impaired osteoblast and osteoclast function (Syberg et al., 2012). The difference may reflect developmental compensation in knockout animals, pharmacokinetic factors, or off-target and metabolite-mediated effects of clopidogrel (Kuszynski and Lauver, 2022). Local calvarial-defect findings involving the active clopidogrel metabolite further indicate that drug form, exposure route, and adenosine-receptor signaling may influence skeletal responses (Mediero et al., 2016). These findings preclude a simple extrapolation from genetic P2Y_12_ deletion to clinical clopidogrel exposure.

### 3.3 Ticagrelor: reversible P2Y_12_ antagonism and ENT1 inhibition

#### 3.3.1 Dual mechanism

Ticagrelor is a direct-acting, reversible P2Y_12_ antagonist that does not require metabolic activation. In addition to inhibiting platelet P2Y_12_ signaling, it inhibits ENT1-mediated adenosine uptake and may thereby increase extracellular adenosine availability, particularly at sites of tissue injury or hypoxia (Cattaneo et al., 2014; Aungraheeta et al., 2016). This ENT1-related activity distinguishes ticagrelor from clopidogrel and prasugrel and provides a mechanistic basis for investigating its effects on skeletal cells.

#### 3.3.2 Adenosine-mediated skeletal effects

In experimental bone-cell systems, ticagrelor enhanced osteoblast differentiation and mineralization through adenosine A_2_A receptor activation (Mediero et al., 2016). Complementary studies of A_2_A receptor signaling, rather than ticagrelor itself, have demonstrated suppression of RANKL-induced osteoclastogenesis through pathways involving PKA/ERK1/2, NF-κB, and AP-1 (Mediero et al., 2012; 2013; Cheng et al., 2022). A_2_A receptor activation has also been linked to increased Sema3A and reduced Sema4D expression, suggesting a role in osteoblast–osteoclast coupling (Mediero et al., 2018). Together, these findings provide experimental support for adenosine-mediated skeletal responses, although the receptor-level findings should be distinguished from direct evidence of ticagrelor action.

#### 3.3.3 Bone regeneration evidence

In a murine calvarial-defect model, ticagrelor delivered locally through calcium phosphate–hydroxyapatite scaffolds increased new bone formation, and this increase was absent in A_2_A receptor-deficient mice (Mediero et al., 2016). In a rat peri-implant model, ticagrelor-containing dual antiplatelet therapy did not significantly impair guided bone regeneration or alter systemic bone-metabolism markers (Kobat et al., 2023); this finding indicates no detectable impairment under the tested conditions rather than a regenerative benefit. Neither model evaluated systemic ticagrelor monotherapy during mechanically loaded long-bone fracture healing.

#### 3.3.4 Clinical pharmacodynamic and skeletal evidence

Human pharmacodynamic studies support, but do not consistently confirm, an adenosine-related effect of ticagrelor. In patients with non-ST-elevation acute coronary syndrome, ticagrelor treatment was associated with higher plasma adenosine and cAMP levels than clopidogrel treatment (Li et al., 2017). However, other randomized pharmacodynamic studies found no consistent differences in circulating adenosine or cellular adenosine uptake between ticagrelor and other P2Y_12_ inhibitors (Ariotti et al., 2018). These pharmacodynamic findings cannot be interpreted as evidence of skeletal benefit.

Human skeletal evidence is limited to one retrospective propensity score–matched cohort in which ticagrelor-based DAPT was associated with a lower risk of major osteoporotic fracture than clopidogrel-based DAPT (Lui et al., 2024). However, residual confounding, concomitant aspirin exposure, and the absence of bone mineral density, bone-turnover, fracture-healing, and implant-related outcomes preclude causal or drug-specific interpretation. No prospective study has evaluated skeletal outcomes associated with ticagrelor treatment.

### 3.4 Prasugrel: irreversible P2Y_12_ antagonism with limited skeletal evidence

Prasugrel is a third-generation thienopyridine that, like clopidogrel, irreversibly inhibits the P2Y_12_ receptor but differs in pharmacokinetics and metabolic activation (Eikelboom et al., 2012). After oral administration, it is rapidly converted to its active metabolite, resulting in faster and more consistent platelet inhibition than clopidogrel, which requires CYP2C19-dependent activation and shows substantial interindividual variability (Mega and Simon, 2015; Kamran et al., 2021). In the TRITON-TIMI 38 trial, prasugrel reduced ischemic cardiovascular events compared with clopidogrel but increased major bleeding, with an unfavorable net clinical profile in patients with prior stroke or transient ischemic attack (Wiviott et al., 2007).

Direct skeletal evidence for prasugrel remains extremely limited. In one rat peri-implant defect study, aspirin combined with prasugrel did not significantly alter bone filling or systemic bone-metabolism markers compared with controls (Kobat et al., 2023). However, this isolated combination model does not establish the skeletal effects of prasugrel monotherapy or its influence on human long-bone fracture healing. No clinical study has evaluated bone mineral density, fracture risk, fracture healing, or implant integration specifically in prasugrel-treated patients. Its clinical skeletal profile therefore remains unknown.

### 3.5 Other clinically relevant antiplatelet agents

Cangrelor is an intravenous, reversible P2Y_12_ antagonist with rapid onset and offset, used during PCI and studied for perioperative bridging. No direct studies were identified evaluating its effects on bone metabolism, fracture risk, or fracture healing; therefore, its skeletal profile remains unknown (Vaduganathan et al., 2017; Salgia et al., 2023).

Cilostazol inhibits phosphodiesterase 3 and increases intracellular cAMP. Murine femoral fracture models, including aged animals, reported enhanced angiogenesis, callus formation, and biomechanical healing (Herath et al., 2015; Menger et al., 2024), whereas a rat study found no significant improvement in clinical, radiographic, or histological healing (Alıç et al., 2016). Thus, its skeletal evidence remains inconsistent and entirely preclinical.

Dipyridamole inhibits cellular adenosine uptake and may increase extracellular adenosine signaling. Local delivery on three-dimensionally printed bioactive ceramic scaffolds enhanced bone formation in a skeletally mature sheep calvarial-defect model (Bekisz et al., 2018), but these findings cannot be extrapolated to systemic antiplatelet therapy. A population-based study associated oral dipyridamole with increased overall, but not osteoporotic, fracture risk, although residual confounding cannot be excluded (Vestergaard et al., 2012). Overall, evidence for cangrelor, cilostazol, and dipyridamole remains insufficient to inform skeletal-based antiplatelet selection. Table 1 distinguishes preclinical findings from clinical skeletal evidence and summarizes overall confidence, key sources of heterogeneity, and translational limitations across individual agents and DAPT combinations.

**TABLE 1:** Evidence map of antiplatelet agents in bone metabolism and fracture-related outcomes.

Agent/strategy	Preclinical evidence	Clinical evidence	Overall confidence and interpretation	Key limitations and heterogeneity	Representative references
Aspirin	Strong. Multiple cell and animal studies demonstrate dose-, route-, and duration-dependent effects. Local or low-concentration exposure has produced osteogenic signals, whereas higher systemic exposure has delayed repair in selected models	Moderate. Observational associations with BMD and fracture risk are inconsistent. The randomized ASPREE trial did not demonstrate fracture prevention with daily low-dose aspirin, and direct human fracture-union evidence remains limited	Moderate. Skeletal effects are context-dependent, and no consistent clinical fracture-prevention or fracture-healing benefit has been established	Dose, route, and duration; age and fall risk; baseline osteoporosis; cardiovascular confounding; heterogeneous endpoints ranging from BMD to fracture incidence and union	(Lack et al., 2013; Barker et al., 2020 ; 2022; de Souza Rendohl et al., 2021)
Clopidogrel	Strong but model-dependent. Selected studies report impaired osteoblast function and trabecular bone loss, whereas reparative responses were observed in a periodontitis model and with local delivery of the active metabolite in calvarial defects. Effects vary by drug form, exposure route, and model	Conflicting. A nationwide cohort suggested exposure-dependent and potentially biphasic fracture associations, whereas adjusted analyses in stroke or TIA populations reported neutral or apparently protective associations. Prospective BMD and fracture-healing studies are unavailable	Low. Human skeletal effects remain uncertain, and current evidence does not justify avoiding clopidogrel on skeletal grounds	Cumulative exposure; cardiovascular indication; stroke-related immobility and falls; baseline bone health; concomitant therapy; CYP2C19-related variability in drug activation	(Jørgensen et al., 2012 ; 2017; Syberg et al., 2012; Vestergaard et al., 2012; Coimbra et al., 2015; Mediero et al., 2016; Kuszynski and Lauver, 2022)
Ticagrelor	Strong but model-specific. ENT1 inhibition and increased extracellular adenosine provide mechanistic plausibility. Osteogenic and anti-osteoclast signals have been demonstrated *in vitro* and following local ticagrelor delivery through calcium phosphate–hydroxyapatite scaffolds in murine calvarial defects	Minimal. One retrospective propensity-matched cohort associated ticagrelor-based DAPT with lower major osteoporotic fracture risk than clopidogrel-based DAPT. No prospective study has assessed BMD, bone turnover, fracture union, nonunion, or implant integration. Human pharmacodynamic studies did not evaluate skeletal outcomes	Low. Findings remain hypothesis-generating, and no clinical skeletal advantage over clopidogrel or prasugrel has been established	Local versus systemic exposure; calvarial versus mechanically loaded long-bone healing; intramembranous versus endochondral ossification; species and dose equivalence; predominantly healthy animal models; residual confounding in the retrospective cohort	(Cattaneo et al., 2014; Mediero et al., 2016; Li et al., 2017; Ariotti et al., 2018; Lui et al., 2024)
Prasugrel	Limited and indirect. A rat peri-implant study found no significant change in bone filling or systemic bone-metabolism markers with aspirin plus prasugrel. Dedicated prasugrel monotherapy studies remain lacking	Minimal. No direct study assessing BMD, fracture risk, fracture healing, or implant-related outcomes was identified. Available cardiovascular trials did not include skeletal endpoints	Very low. Its skeletal profile remains unknown, and skeletal effects cannot be inferred from antiplatelet potency or class membership	Combination therapy prevents isolation of prasugrel-specific effects; absence of dedicated monotherapy studies and direct human skeletal endpoints	(Eikelboom et al., 2012; Ferri et al., 2013; Mega and Simon, 2015; Kobat et al., 2023)
Cangrelor	Absent. No direct bone-cell, skeletal-remodeling, or fracture-healing study was identified	Minimal. PCI and bridging studies evaluate platelet inhibition, ischemic events, and bleeding rather than skeletal outcomes	Very low. Its skeletal effects remain unknown	Short-term intravenous exposure; absence of dedicated skeletal endpoints; bridging populations differ substantially from chronic antiplatelet users	(Vaduganathan et al., 2017; Salgia et al., 2023)
Cilostazol	Moderate but conflicting. Mouse fracture models, including aged animals, reported enhanced angiogenesis, callus formation, and biomechanical healing, whereas a rat study found no significant improvement in radiographic or histological fracture healing	Minimal. No direct human study evaluating BMD, fracture risk, or fracture healing was identified	Very low. Findings remain entirely preclinical and do not establish a clinical fracture-healing benefit	Species, dose, age, and fracture-model differences; inconsistent animal findings; absence of cardiovascular comorbidity and prospective human validation	(Herath et al., 2015; Alıç et al., 2016; Menger et al., 2024)
Dipyridamole	Moderate but model-specific. Local delivery on three-dimensionally printed bioactive ceramic scaffolds promoted bone formation in a skeletally mature sheep calvarial-defect model through mechanisms consistent with increased extracellular adenosine signaling	Minimal. One population-based study associated oral dipyridamole with increased overall, but not osteoporotic, fracture risk. No clinical fracture-healing study is available	Low. Local osteogenic findings do not establish a systemic skeletal benefit, and clinical effects remain uncertain	Local scaffold delivery versus systemic oral therapy; model-specific exposure; observational confounding; differences in fracture definitions and baseline skeletal risk	(Vestergaard et al., 2012; Bekisz et al., 2018)
DAPT combinations	Limited. Peri-implant animal evidence suggests that skeletal effects may depend on aspirin dose, the paired P2Y_12_ inhibitor, treatment duration, and the experimental model	Minimal and indirect. Most orthopedic DAPT studies evaluate bleeding, transfusion, or surgical timing rather than skeletal outcomes. The retrospective ticagrelor–clopidogrel comparison was conducted within aspirin-based DAPT and cannot fully isolate the contribution of the P2Y_12_ inhibitor	Very low. The net skeletal effect of DAPT remains unknown and should not influence guideline-directed antiplatelet treatment	Heterogeneous drug combinations and treatment durations; confounding by cardiovascular indication; inability to separate component-drug effects; absence of prospective healing endpoints	(Kobat et al., 2023; Lu et al., 2024b; Lui et al., 2024)

Evidence was assessed qualitatively according to its volume, directness, consistency, and clinical applicability. Strong denotes multiple direct lines of evidence; moderate, several studies with limitations in consistency or breadth; limited, few studies or predominantly indirect evidence; minimal, sparse clinical evidence; and absent, no direct evidence identified. Conflicting denotes discordant findings. Model-specific, model-dependent, and context-dependent qualify the scope or variability of findings rather than constitute separate evidence levels. Minimal clinical evidence may include isolated studies with skeletal endpoints or indirect pharmacodynamic and perioperative studies, as specified in each row; the latter were not considered direct skeletal evidence. Overall confidence reflects the evidence supporting conclusions about human skeletal effects, considering model relevance, species and exposure equivalence, confounding, and prospective clinical validation. These categories do not indicate the direction or magnitude of skeletal benefit and are not a formal GRADE, assessment. References are representative rather than exhaustive. No category supports antiplatelet selection or switching on skeletal grounds. BMD, bone mineral density; DAPT, dual antiplatelet therapy; ENT1, equilibrative nucleoside transporter 1; PCI, percutaneous coronary intervention; TIA, transient ischemic attack.

## 4 Effects of antiplatelet agents on fracture healing: preclinical and clinical evidence

### 4.1 Biological basis of fracture healing and purinergic signaling

Fracture healing is a tightly regulated regenerative process involving inflammation, cell recruitment, callus formation, and remodeling, coordinated by platelets, immune cells, MSCs, osteoblasts, osteoclasts, and endothelial cells (Bartold and Ivanovski, 2025). After injury, platelets form a hematoma and release growth factors such as PDGF, TGF-β, and VEGF, together with ECM proteins that establish a provisional scaffold and strongly stimulate bone cell proliferation, matrix deposition, and angiogenesis (Gruber et al., 2002). Platelet-derived signals also promote periosteal activation and osteoclastogenesis, supporting early repair (Al-Hamed et al., 2021).

Purinergic signaling is central to this process. Injury-induced release of ATP and ADP activates P2 receptors on bone and immune cells, while ATP degradation via CD39/CD73 generates adenosine that engages P1 receptors (Agrawal and Jørgensen, 2021). P2Y_2_-mediated signaling promotes osteoblast differentiation, BMP expression, and mechanotransduction via PI3K/AKT and ERK1/2 pathways (Ayala-Peña et al., 2013; Xing et al., 2021). In contrast, adenosine A2A receptor activation enhances osteoblast activity, mineralization, and angiogenesis while suppressing osteoclastogenesis, supporting fracture repair (Mediero et al., 2016; Newman and Varghese, 2023).

ATP/ADP–P2 and adenosine–P1 signaling participate in fracture repair and provide mechanistically plausible, but clinically unvalidated, interaction points for antiplatelet therapy (Newman and Varghese, 2023). As summarized in Figure 3, these potential interactions span the inflammatory, callus-forming, mineralization, and remodeling phases, but stage-specific clinical effects remain unestablished.

**FIGURE 3:**
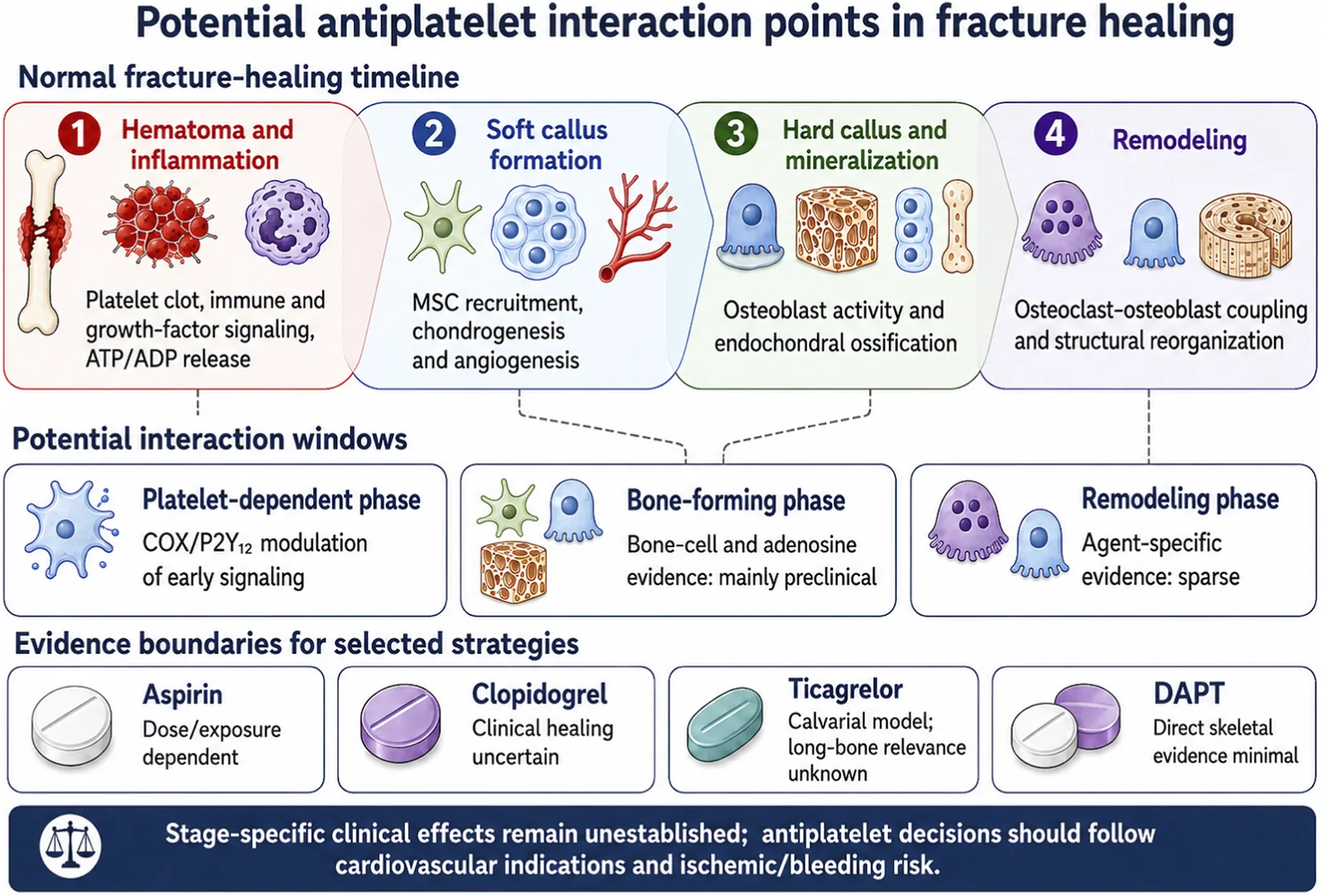
Potential antiplatelet interaction points in fracture healing. Fracture healing proceeds through overlapping hematoma/inflammatory, soft-callus, hard-callus/mineralization, and remodeling phases involving platelets, immune cells, mesenchymal stromal cells, osteoblasts, osteoclasts, and endothelial cells (Gruber et al., 2002; Bartold and Ivanovski, 2025). Antiplatelet therapy may interact with early platelet-dependent signaling through COX or P2Y_12_ inhibition, whereas bone-cell and adenosine-related findings during callus formation and mineralization derive mainly from preclinical models. Aspirin findings are exposure dependent; clopidogrel evidence remains primarily preclinical; ticagrelor bone-regeneration evidence is based mainly on local delivery in a murine calvarial-defect model rather than systemic long-bone fracture healing; and direct DAPT skeletal evidence remains sparse (Lack et al., 2013; Syberg et al., 2012; Mediero et al., 2016; Marmor et al., 2020). Dashed connectors indicate potential interaction windows rather than established clinical effects. Current evidence does not support modifying antiplatelet therapy on stage-specific skeletal grounds.

### 4.2 Aspirin and fracture healing

Aspirin may affect experimental bone repair through cyclooxygenase inhibition and altered prostaglandin signaling, with effects that vary according to dose and route of administration (Xie et al., 2019). In a rabbit ulnar osteotomy model, aspirin delayed healing in a dose-dependent manner, with a threshold approximating a human dose of 325 mg (Lack et al., 2013). Other experimental studies using local or lower-dose exposure have reported more favorable bone-formation responses, emphasizing the importance of exposure conditions (de Souza Rendohl et al., 2021). In a tooth-extraction model, aspirin impaired alveolar bone healing and altered osteoclast-related remodeling (Wang et al., 2024). Clinical evidence regarding fracture healing remains limited. The ASPREE trial evaluated fracture incidence rather than fracture healing and found no reduction in fracture risk with daily low-dose aspirin (Barker et al., 2022). Overall, aspirin effects on experimental bone repair appear exposure dependent, but their clinical relevance remains uncertain and should not alter guideline-based perioperative management.

### 4.3 Clopidogrel and fracture healing

Clopidogrel exerts complex effects on fracture healing through both P2Y_12_-dependent and potential off-target mechanisms. *In vitro*, clopidogrel dose-dependently inhibits osteoblast mineralization, alkaline phosphatase activity, collagen formation, and cell proliferation, while promoting adipogenic differentiation of mesenchymal stem cells (Syberg et al., 2012). It also suppresses osteoclast formation and resorptive activity, suggesting overall suppression of bone turnover (Syberg et al., 2012). *In vivo*, clopidogrel-treated mice showed reduced bone mineral density and marked trabecular bone loss, particularly in trabecular-rich skeletal compartments (Syberg et al., 2012). Mechanistically, discrepancies between genetic P2Y_12_ deletion and pharmacologic inhibition suggest possible metabolite-mediated or P2Y_12-_independent effects (Kuszynski and Lauver, 2022). Epidemiological studies remain conflicting, with some reporting increased fracture risk during long-term treatment and others showing neutral or protective associations after adjustment for comorbidities (Jørgensen et al., 2012). Importantly, clinical studies in hip fracture patients indicate that early surgery while continuing clopidogrel does not significantly increase mortality or major bleeding complications (Lu W. et al., 2024). Overall, preclinical data raise concerns that clopidogrel may impair fracture repair and bone regeneration, although definitive clinical evidence remains lacking (Kuszynski and Lauver, 2022).

### 4.4 Ticagrelor and bone regeneration

Preclinical studies have examined ticagrelor in calvarial and peri-implant bone-regeneration models. In murine calvarial defects, locally delivered ticagrelor increased new bone formation (Mediero et al., 2016). In a rat peri-implant model, ticagrelor-containing dual antiplatelet therapy did not significantly impair guided bone regeneration, although this finding does not demonstrate a regenerative benefit (Kobat et al., 2023). These studies assessed local scaffold delivery or combination therapy in defect models rather than systemic ticagrelor monotherapy during mechanically loaded long-bone fracture healing. No clinical study has evaluated fracture union or orthopedic bone regeneration in ticagrelor-treated patients.

### 4.5 Dual antiplatelet therapy combinations

Direct skeletal evidence for dual antiplatelet therapy remains limited. In a rat peri-implant defect model, aspirin combined with clopidogrel, prasugrel, or ticagrelor did not significantly alter bone filling or systemic bone-metabolism markers compared with controls (Kobat et al., 2023). In a tooth-extraction model, combined aspirin and clopidogrel produced alveolar-healing responses more similar to clopidogrel alone than to aspirin alone, suggesting nonadditive effects in this specific experimental setting (Wang et al., 2024). However, neither model establishes effects on mechanically loaded long-bone fracture healing. In one retrospective propensity score–matched cohort, ticagrelor-based DAPT was associated with a lower risk of major osteoporotic fracture than clopidogrel-based DAPT, but residual confounding precludes causal interpretation (Lui et al., 2024). No clinical study has compared fracture union, delayed union, nonunion, or implant integration according to DAPT composition. Current evidence is therefore insufficient to support modification of DAPT regimens on skeletal grounds.

### 4.6 Guided bone regeneration around implants

Guided bone regeneration (GBR) models suggest divergent skeletal effects among antiplatelet agents. In a rat peri-implant defect model, aspirin alone or combined with clopidogrel, prasugrel, or ticagrelor did not significantly alter bone filling ratios or biochemical markers of bone metabolism after 8 weeks, indicating that dual antiplatelet therapy (DAPT) did not impair GBR (Kobat et al., 2023). However, continuous clopidogrel treatment unexpectedly enhanced titanium implant osseointegration in rabbits, increasing bone-to-implant contact, trabecular thickness, and peri-implant bone density compared with controls (Lillis et al., 2024). Clinical observational studies similarly found no significant increase in early implant failure or perioperative bleeding in patients continuing aspirin or clopidogrel during dental implantation (Tabrizi et al., 2018; Jonas et al., 2024). In extraction socket models, clopidogrel increased BV/TV, trabecular thickness, and the OPG/RANKL ratio, whereas aspirin impaired alveolar bone healing (Wang et al., 2024).

### 4.7 Translational limitations: from preclinical models to human fracture healing

Translating preclinical skeletal findings to human fracture healing requires careful consideration of drug delivery and repair biology. For ticagrelor, local delivery through calcium phosphate–hydroxyapatite scaffolds in murine calvarial defects creates an exposure setting distinct from conventional systemic therapy (Mediero et al., 2016). Calvarial defects are non-load-bearing and heal predominantly through intramembranous ossification, whereas long-bone fracture healing depends on the mechanical environment and commonly involves endochondral callus formation (Marmor et al., 2020). Increased bone formation in a calvarial defect therefore cannot be equated with improved union in a mechanically loaded long bone.

Additional barriers include species differences in bone architecture, turnover, healing kinetics, and drug metabolism, together with uncertainty about whether experimental doses and tissue concentrations reflect clinically relevant systemic exposure (Bigham-Sadegh and Oryan, 2015; Lopas et al., 2023). Most experimental models use young, otherwise healthy animals and do not reproduce the aging, osteoporosis, cardiovascular comorbidity, and concomitant therapies common in patients receiving antiplatelet treatment (Marmor et al., 2020; Nafisi et al., 2025). These differences limit extrapolation across populations and treatment settings and highlight the need for exposure-relevant models with functional outcomes, including mechanical strength and fracture union.

### 4.8 Sources of heterogeneity and conflicting results

Conflicting findings across aspirin, clopidogrel, and fracture studies likely reflect differences in drug exposure, study populations, and endpoints. Aspirin effects vary with dose, route, and duration: local or low-concentration exposure may support osteogenic responses, whereas higher systemic exposure may interfere with bone repair. Favorable observational associations were not confirmed in the randomized ASPREE trial (Lack et al., 2013; Barker et al., 2020; 2022). Clopidogrel findings may also depend on cumulative exposure; a nationwide cohort reported a biphasic association and higher fracture risk after more than 1 year, whereas adjusted analyses in stroke or transient ischemic attack populations were neutral or apparently protective (Jørgensen et al., 2012; 2017).

Age, cardiovascular indication and comorbidity, immobility, fall risk, baseline osteoporosis or BMD, and concomitant treatments may influence both drug selection and skeletal outcomes (Jørgensen et al., 2012; 2017; Lui et al., 2024). Comparisons are further limited by heterogeneous endpoints, ranging from cell and animal outcomes to BMD, incident fracture, and fracture union. CYP2C19 variants alter clopidogrel activation and platelet response, but their relevance to skeletal outcomes remains unknown (Thomas et al., 2023). These factors preclude direct agent-ranking conclusions.

## 5 Perioperative antiplatelet management in orthopedic surgery: balancing thrombotic, bleeding, and skeletal risks

### 5.1 Current guideline recommendations

The 2024 AHA/ACC guideline provides an updated framework for perioperative cardiovascular management in noncardiac surgery, complemented by the 2022 ACCP/CHEST recommendations on antithrombotic therapy (Douketis et al., 2022; Thompson et al., 2024). When P2Y_12_ inhibitor interruption is necessary, the 2024 guideline lists intervals to restoration of platelet function of 5–7 days for clopidogrel, 3–5 days for ticagrelor, and 7–10 days for prasugrel (Thompson et al., 2024). The 2022 ACCP/CHEST guideline suggests preoperative interruption for 5 days, 3–5 days, and 7 days, respectively (Douketis et al., 2022). These intervals apply when interruption has been judged appropriate and should not be interpreted as a requirement to discontinue therapy in every patient.

The 2022 ACCP/CHEST guideline generally favors aspirin continuation over interruption for elective noncardiac surgery (Douketis et al., 2022). Management in patients with coronary stents requires additional consideration of the timing and indication for PCI, as discussed below. Interrupted antiplatelet therapy should be resumed promptly once adequate hemostasis permits, with timing individualized according to postoperative bleeding and thrombotic risks (du Breuil, 2023).

### 5.2 Timing of surgery after PCI

For elective noncardiac surgery requiring interruption of at least one antiplatelet agent, the 2024 AHA/ACC guideline advises an ideal delay of ≥12 months after drug-eluting stent (DES) implantation for acute coronary syndrome and considers a delay of ≥6 months reasonable after DES implantation for chronic coronary disease. Time-sensitive surgery requiring interruption may be considered ≥3 months after DES implantation when the consequences of delay outweigh perioperative cardiovascular risk (Thompson et al., 2024).

Elective surgery requiring antiplatelet interruption within 30 days of bare-metal stent (BMS) or DES implantation is considered potentially harmful. For time-sensitive surgery within 30 days of BMS implantation or <3 months after DES implantation, DAPT should be continued unless bleeding risk outweighs the benefit of preventing stent thrombosis (Thompson et al., 2024). In orthopedic practice, surgical urgency and the consequences of delaying fracture treatment should therefore be considered alongside stent-related risk through multidisciplinary assessment.

### 5.3 Aspirin continuation in patients with coronary stents

Current guidelines generally support perioperative aspirin continuation in patients with coronary stents undergoing noncardiac surgery because interruption may increase the risk of myocardial infarction and stent thrombosis (Thompson et al., 2024). The POISE-2 subgroup analysis in patients with prior PCI suggested reductions in perioperative myocardial infarction and the composite of death or nonfatal myocardial infarction with aspirin, while its effect on major or life-threatening bleeding remained uncertain (Douketis et al., 2022; Douketis and Spyropoulos, 2023). Observational studies similarly associate antiplatelet interruption with higher rates of adverse cardiovascular events (Douketis et al., 2022). However, recent data from the ASSURE-DES trial suggest that in stable patients more than 1 year after DES implantation, temporary aspirin discontinuation may not significantly increase ischemic events, although minor bleeding is reduced (Kang et al., 2024). Current practice therefore emphasizes individualized risk assessment according to stent type, timing since PCI, and surgical bleeding risk (Douketis and Spyropoulos, 2023). When P2Y_12_ inhibitor interruption is appropriate, aspirin is generally maintained if bleeding risk permits; patients within the early post-stenting periods described in Section 5.2 may instead require continued DAPT (Thompson et al., 2024). Multidisciplinary decision-making remains essential (Levine et al., 2016; Capodanno et al., 2018).

### 5.4 Skeletal outcomes as an evidence gap

Current perioperative antiplatelet guidelines focus primarily on thrombotic and bleeding risks while largely overlooking potential skeletal effects, despite growing evidence that antiplatelet agents influence bone remodeling (Mediero et al., 2016). P2Y_12_ signaling regulates osteoclast and osteoblast activity, and experimental studies suggest that its inhibition may alter bone metabolism (Su et al., 2012). Notably, clopidogrel has shown adverse skeletal findings in selected preclinical studies and conflicting fracture-risk associations in observational cohorts (Syberg et al., 2012). In contrast, selected preclinical studies have linked ticagrelor-associated ENT1 inhibition and A2AR signaling to osteogenic responses, although human skeletal evidence is limited to one retrospective fracture-risk cohort, with no prospective BMD, bone-turnover, fracture-healing, or implant-related studies available (Mediero et al., 2016; Lui et al., 2024). Aspirin also appears to have dose-dependent skeletal effects, with low doses potentially benefiting bone while higher doses may impair fracture healing (Xie et al., 2019). Despite these findings, perioperative studies and guidelines rarely evaluate fracture healing, nonunion, or functional skeletal recovery (Abdulhamid, 2020; Tang et al., 2025). Future orthopedic studies should evaluate skeletal outcomes alongside thrombotic and bleeding outcomes before these considerations are incorporated into clinical recommendations.

### 5.5 Bridging strategies

The 2024 AHA/ACC guideline allows consideration of intravenous antiplatelet bridging in selected patients at high thrombotic risk when noncardiac surgery cannot be deferred, particularly within 6 months of DES implantation or 30 days of BMS implantation (Thompson et al., 2024). This selective approach should be distinguished from routine bridging, which the 2022 ACCP/CHEST guideline advises against in patients with coronary stents requiring antiplatelet interruption (Douketis et al., 2022).

Cangrelor is a potential bridging option because of its rapid onset and offset. The BRIDGE trial demonstrated maintenance of platelet inhibition without a significant increase in excessive CABG-related bleeding, but its cardiac-surgery setting limits direct application to orthopedic procedures (Sullivan et al., 2021). Observational experience provides additional information, although both bleeding and ischemic events have been reported (Salgia et al., 2023). Bridging should therefore be reserved for selected cases after multidisciplinary assessment; its effects on orthopedic skeletal outcomes remain unstudied.

## 6 Skeletal considerations during antiplatelet therapy: a hypothesis-generating framework

### 6.1 Current clinical priorities and the skeletal evidence gap

Current antiplatelet selection is based primarily on cardiovascular indications, ischemic risk, and bleeding risk, and skeletal outcomes are not incorporated into existing treatment guidelines (Passacquale et al., 2022; Virani et al., 2023). The coexistence of cardiovascular disease and skeletal fragility therefore supports concurrent bone-health assessment, but not agent-specific antiplatelet selection. Patients with osteoporosis or prior fragility fractures require guideline-based bone-health assessment and management, including fracture-risk evaluation when appropriate (Ye et al., 2025), whereas those undergoing active fracture healing represent a priority population for prospective investigation of antiplatelet-related skeletal outcomes. Stage-specific effects of antiplatelet agents on human fracture healing remain unknown. Overall, the available evidence remains hypothesis-generating and supports prospective evaluation of skeletal endpoints rather than modification of current antiplatelet practice.

### 6.2 High-priority patient populations

Several patient groups warrant priority consideration for concurrent bone-health assessment and prospective skeletal outcome research. Elderly patients with hip fractures and coronary stents require balancing thrombotic, bleeding, and skeletal risks. A recent meta-analysis found that continuation of antiplatelets during hip fracture surgery did not significantly increase transfusion requirements, while earlier surgery reduced hospital stay without increasing mortality (Tang et al., 2025). Patients with recent coronary stents face substantial cardiovascular risk if dual antiplatelet therapy is interrupted, and perioperative aspirin continuation may reduce myocardial infarction despite a modest increase in bleeding (Douketis et al., 2022).

Patients with diabetes and chronic kidney disease (CKD) are also high-priority populations because of impaired fracture healing, increased fracture risk, and high cardiovascular burden. Antiplatelet therapy reduces cardiovascular events but may increase bleeding, while CKD can alter responsiveness to some agents (Smilowitz and Berger, 2020). Similarly, CKD patients experience markedly elevated risks of both myocardial infarction and skeletal complications, necessitating individualized treatment decisions (Patrono et al., 2017).

Additional populations include patients undergoing joint arthroplasty, vertebral fractures, delayed unions or nonunions, multiple fragility fractures, skeletal metastases, postmenopausal women, inflammatory arthritis, and chronic glucocorticoid users. These populations should be prioritized for prospective skeletal outcome studies, but current evidence does not support altering antiplatelet selection on skeletal grounds.

### 6.3 Ticagrelor: preclinical osteogenic signals and the clinical evidence gap

Ticagrelor’s preclinical osteogenic signals warrant further investigation, but their relevance to clinical treatment decisions remains uncertain. The retrospective association between ticagrelor-based DAPT and lower major osteoporotic fracture risk provides a preliminary clinical signal, although residual confounding and concomitant aspirin exposure prevent attribution of this finding to ticagrelor alone (Lui et al., 2024). Moreover, fracture-risk associations do not establish benefits for fracture healing or implant integration. No prospective study has established a clinical skeletal advantage over clopidogrel or prasugrel. Accordingly, skeletal considerations should prompt appropriate bone-health assessment and management rather than selection of, or switching to, ticagrelor; antiplatelet choice should remain guided by cardiovascular indications and ischemic and bleeding risks.

### 6.4 Proposed framework for bone-health assessment and research

Antiplatelet management should remain based on cardiovascular indications and established ischemic and bleeding-risk assessment. Current cardiovascular guidelines do not incorporate skeletal parameters into antiplatelet selection (Passacquale et al., 2022; Virani et al., 2023). Nevertheless, FRAX, prior fragility fractures, active fracture healing, planned orthopedic surgery, glucocorticoid exposure, and chronic inflammatory disease may identify patients requiring guideline-based bone-health evaluation and populations that should be prioritized for prospective skeletal outcome research (Ye et al., 2025).

The cardiovascular indication remains the primary determinant of antiplatelet intensity and duration. In acute coronary syndromes, dual antiplatelet therapy is generally required, whereas chronic coronary disease or peripheral artery disease may allow single antiplatelet therapy or selected P2Y_12_ inhibitor monotherapy (Bonaca et al., 2024). Current evidence does not support selecting or switching to ticagrelor, or avoiding clopidogrel, on the basis of presumed skeletal effects. Antiplatelet choice should remain determined by cardiovascular indications, ischemic protection, bleeding risk, contraindications, drug interactions, and current guideline recommendations. In patients with osteoporosis, prior fragility fractures, or active bone healing, skeletal risk should instead prompt guideline-based bone-health assessment and management rather than unvalidated antiplatelet substitution. Accordingly, Figure 4 presents a hypothesis-generating framework for concurrent, guideline-based bone-health assessment and future research, rather than a basis for agent-specific antiplatelet selection or switching.

**FIGURE 4:**
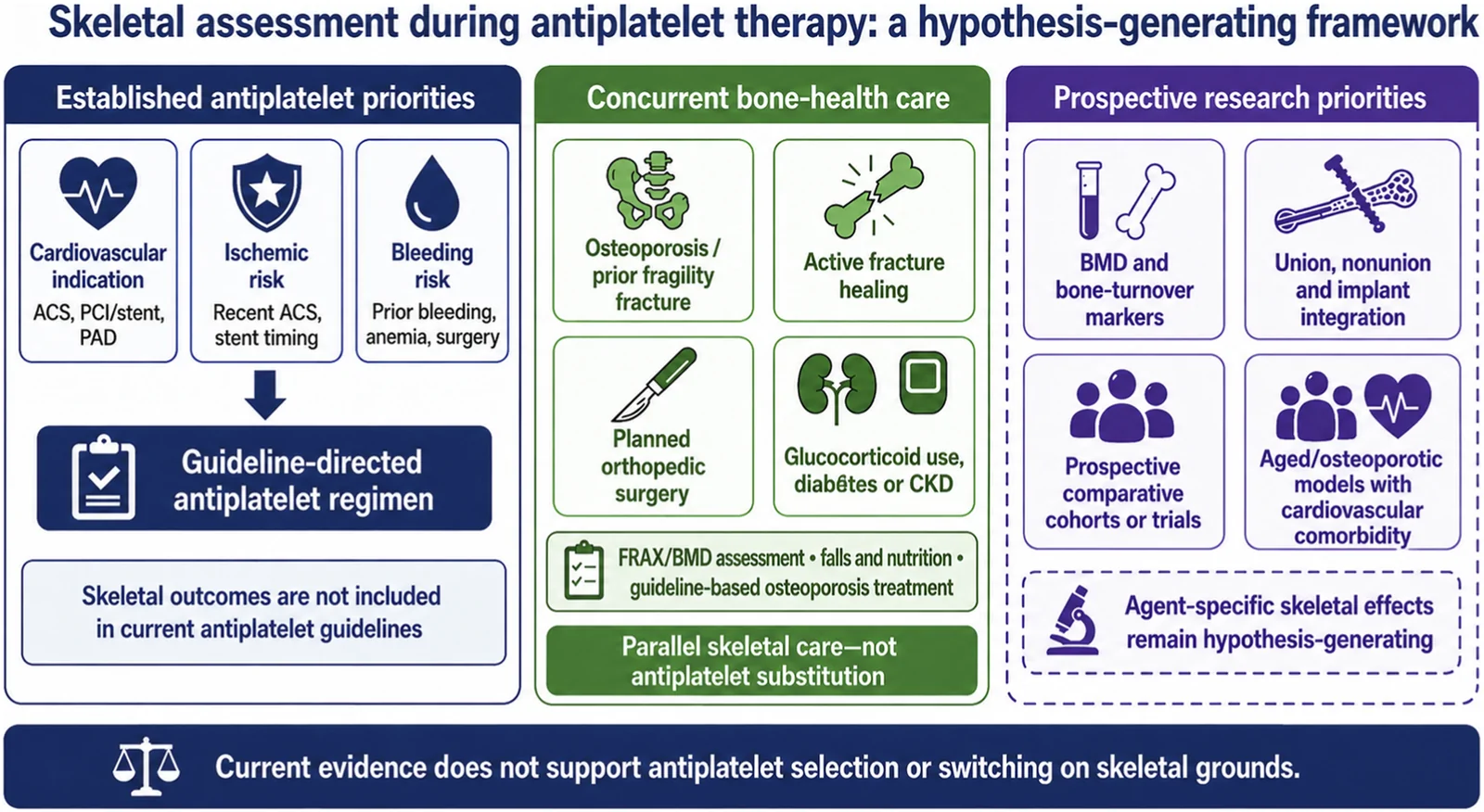
Skeletal assessment during antiplatelet therapy: a hypothesis-generating framework. Current antiplatelet regimens should remain determined by cardiovascular indications, ischemic risk, bleeding risk, contraindications, drug interactions, and guideline recommendations; skeletal outcomes are not incorporated into current antiplatelet guidelines (Passacquale et al., 2022; Virani et al., 2023; Bonaca et al., 2024). Osteoporosis, prior fragility fracture, active fracture healing, planned orthopedic surgery, glucocorticoid use, diabetes, and chronic kidney disease may warrant concurrent guideline-based bone-health assessment and management, including FRAX or BMD evaluation, fall prevention, nutritional optimization, and osteoporosis treatment where indicated (Ye et al., 2025). These measures represent a parallel care pathway and do not justify antiplatelet substitution. Prospective studies should evaluate BMD, bone-turnover markers, fracture union, nonunion, and implant integration. Agent-specific skeletal effects remain hypothesis-generating, and current evidence does not support antiplatelet selection or switching on skeletal grounds. BMD, bone mineral density; FRAX, Fracture Risk Assessment Tool.

### 6.5 Limitations and caveats

Several limitations should temper interpretation of the skeletal evidence during antiplatelet therapy. Preclinical findings may not translate clinically, as aspirin-related osteoblast and osteoclast effects observed experimentally were not confirmed by the ASPREE trial, which found no fracture-risk reduction with low-dose aspirin in healthy older adults (Barker et al., 2022). Observational studies are vulnerable to confounding by cardiovascular disease, immobility, inflammation, and comorbidities, helping explain conflicting clopidogrel findings across populations (Jørgensen et al., 2017). Prospective randomized trials comparing P2Y_12_ inhibitors for fracture risk, fracture healing, bone mineral density, or bone turnover markers are lacking, and existing evidence mainly derives from post-hoc analyses, registries, and mechanistic studies (Barker et al., 2020; Mahmood et al., 2021). Moreover, ticagrelor-related preclinical osteogenic signals and interindividual pharmacokinetic variability require clinical validation (Thomas et al., 2023).

Overall, the proposed framework is hypothesis-generating rather than practice-changing. Cardiovascular indications and ischemic and bleeding risks should remain the basis of antiplatelet treatment. Skeletal risk should prompt guideline-based bone-health assessment and management, as well as prospective investigation, rather than antiplatelet selection or substitution.

## 7 Future directions

### 7.1 Prospective skeletal outcome studies

Prospective skeletal outcome studies are needed to clarify whether different antiplatelet agents exert clinically meaningful effects on bone metabolism and fracture repair. Current cardiovascular trials have rarely prespecified bone mineral density, fracture incidence, or bone turnover markers as endpoints, leaving uncertainty about the skeletal consequences of long-term P2Y_12_ inhibition (Passacquale et al., 2022). Future trials should move beyond post-hoc safety reporting and directly compare ticagrelor, clopidogrel, and prasugrel in patients with high skeletal risk, using dual-energy X-ray absorptiometry at baseline and during follow-up. Standardized bone turnover marker panels, particularly procollagen type I N-terminal propeptide and C-terminal cross-linking telopeptide of type I collagen, could provide early signals of altered bone formation or resorption (Bhattoa et al., 2025). Orthopedic trauma cohorts should assess time to radiographic union, functional recovery, delayed union, and nonunion, with emerging fracture-repair biomarkers incorporated when feasible (Working et al., 2025). Future protocols should stratify patients by FRAX score, prior fragility fracture, osteoporosis treatment, glucocorticoid exposure, kidney disease, smoking, and fixation method. Ultimately, skeletal endpoints should become planned outcomes rather than incidental safety observations. To move the field from mechanistic plausibility to clinically actionable evidence, future studies should prespecify skeletal endpoints that match the biological question, orthopedic scenario, and expected time course of bone remodeling (Table 2).

**TABLE 2:** Research priorities and candidate skeletal endpoints for future antiplatelet studies.

Research priority	Study population or model	Core outcomes	Design and measurements	Primary objective	Representative references
Long-term skeletal outcomes	Adults receiving long-term aspirin or P2Y_12_ inhibitor therapy, particularly older adults and patients at high fracture risk	Change in BMD; incident fragility and vertebral fractures	Prospective cohorts or trial substudies; baseline and follow-up DXA; vertebral fracture assessment; FRAX; follow-up ≥24 months	Determine whether agent and cumulative exposure are associated with skeletal fragility after adjustment for baseline risk	(Jørgensen et al., 2012 ; 2017; Barker et al., 2022; Lui et al., 2024)
Bone turnover and pharmacodynamic studies	Patients initiating aspirin, clopidogrel, ticagrelor, or prasugrel	P1NP, CTX, BALP, osteocalcin, and TRAP-5b	Standardized fasting serum sampling at baseline and approximately 1, 3, 6, and 12 months	Detect early agent-associated changes in bone formation or resorption without assuming clinical skeletal benefit	(Moghaddam et al., 2011; Brown et al., 2022; Bhattoa et al., 2025)
Fracture-healing cohorts	Patients with long-bone fractures who have an independent cardiovascular indication for antiplatelet therapy	Time to radiographic union; delayed union; nonunion; revision surgery; functional recovery	Prospective multicenter cohorts; serial radiographs; CT when clinically indicated; validated functional measures; follow-up 3–12 months	Evaluate associations between antiplatelet exposure and clinically relevant fracture-healing outcomes	(Marmor et al., 2020; Bartold and Ivanovski, 2025; Working et al., 2025)
Arthroplasty and implant studies	Patients undergoing orthopedic arthroplasty or dental implantation while receiving antiplatelet therapy	Periprosthetic or peri-implant BMD; implant migration; osseointegration; early implant failure; loosening; revision	DXA or RSA where applicable; CT and standardized radiographic or registry-based implant assessments; follow-up 6–24 months	Evaluate bone–implant outcomes while concurrently recording cardiovascular and bleeding events	(Tabrizi et al., 2018; Kobat et al., 2023; Jonas et al., 2024; Lillis et al., 2024)
Agent-specific translational studies	Sequential *in vitro* and *in vivo* studies using clinically relevant systemic exposure, including aged, osteoporotic, and cardiovascular-comorbidity long-bone models; local ticagrelor delivery assessed separately	Drug exposure; platelet response; adenosine signaling; osteoblast and osteoclast activity; callus formation; mechanical strength	PK/PD analysis, micro-CT, histomorphometry, biomechanical testing, and comparison across agents, doses, and delivery routes	Determine whether agent-specific skeletal signals are reproducible, exposure relevant, and translatable to human long-bone healing	(Syberg et al., 2012; Bigham-Sadegh and Oryan, 2015; Mediero et al., 2016; Marmor et al., 2020; Lopas et al., 2023)
Comparative clinical and effect-modifier studies	Patients with cardiovascular indications and coexisting skeletal risk, including osteoporosis, prior fragility fracture, diabetes, or CKD	Skeletal, cardiovascular, and bleeding outcomes assessed separately	Prospective comparative cohorts or pragmatic trials; prespecified dose, duration, adherence, baseline bone status, comorbidities, concomitant therapy, and CYP2C19 genotype where relevant; follow-up 12–36 months	Determine whether agent-specific skeletal associations persist after confounding adjustment and identify potential effect modifiers	(Jørgensen et al., 2012 ; 2017; Thomas et al., 2023; Lui et al., 2024; El Rouby and Johnson, 2025)

This table presents a hypothesis-generating research agenda rather than clinical guidance. Studies should prespecify antiplatelet indication, agent, dose, duration, adherence, age, baseline osteoporosis or BMD, prior fracture, cardiovascular comorbidities, concomitant medications, smoking, and fall risk. CYP2C19 genotype should be considered where relevant to clopidogrel, although its relationship with skeletal outcomes remains unknown. Cardiovascular and bleeding outcomes should be evaluated concurrently with skeletal endpoints. Local drug-delivery findings should be distinguished from conventional systemic exposure, and calvarial-defect findings should not be directly extrapolated to long-bone fracture healing. No proposed study design supports current antiplatelet selection or switching on skeletal grounds. BALP, bone-specific alkaline phosphatase; BMD, bone mineral density; CKD, chronic kidney disease; CT, computed tomography; CTX, C-terminal telopeptide of type I collagen; CYP2C19, cytochrome P450 family 2 subfamily C member 19; DXA, dual-energy X-ray absorptiometry; FRAX, Fracture Risk Assessment Tool; P1NP, procollagen type I N-terminal propeptide; PK/PD, pharmacokinetic/pharmacodynamic; P2Y_12_, purinergic receptor P2Y_12_; RSA, radiostereometric analysis; TRAP-5b, tartrate-resistant acid phosphatase 5b.

### 7.2 Randomized studies in orthopedic patients

Patients receiving antiplatelet therapy during fracture treatment or joint replacement represent an important population for prospective skeletal outcome research. The PREVENT CLOT trial demonstrated that aspirin was noninferior to low-molecular-weight heparin for thromboprophylaxis after extremity or pelvic fractures, but it neither assessed skeletal healing endpoints nor compared P2Y_12_ inhibitors (O’Toole et al., 2023). Its findings therefore cannot establish agent-specific effects on bone repair.

Future studies should evaluate skeletal outcomes prospectively when clinically acceptable antiplatelet options are compared for established cardiovascular indications. Relevant outcomes include radiographic union, delayed union, nonunion, revision surgery, periprosthetic bone loss, implant migration, and osseointegration. Randomized comparisons should be considered only where genuine cardiovascular equipoise exists; otherwise, multicenter prospective cohorts with adjustment for age, osteoporosis, fracture characteristics, fixation method, cardiovascular indication, comorbidities, concomitant therapies, and bleeding risk would be more appropriate. Antiplatelet treatment should not be assigned or modified solely to test presumed skeletal benefits.

### 7.3 Translational studies of ticagrelor

Building on ticagrelor’s ENT1-related pharmacology and the existing local bone-regeneration findings, future studies should relate systemic drug exposure to local adenosine availability and skeletal responses using clinically relevant dosing (Cattaneo et al., 2014; Mediero et al., 2016). Pharmacokinetic and pharmacodynamic measurements should establish whether exposure sufficient for platelet inhibition also alters adenosine signaling at the repair site. Mechanically loaded long-bone fracture models should assess callus formation, radiographic union, and mechanical strength alongside osteoblast and osteoclast activity. These studies should include aged and osteoporotic animals, as well as models incorporating cardiovascular comorbidity and relevant concomitant therapies.

Local delivery strategies should be evaluated separately from systemic treatment, with characterization of drug release, tissue exposure, systemic absorption, and local and systemic safety. Pharmacological or genetic approaches could help distinguish adenosine-receptor-mediated responses from P2Y_12_-dependent effects. Biomarker and transcriptomic analyses may further identify pathway engagement and response heterogeneity, but should complement functional healing outcomes rather than serve as substitutes for them.

### 7.4 Biomarker-based evaluation

Biomarker-based evaluation should become a central component of future antiplatelet skeletal-outcome studies. Serum procollagen type I N-terminal propeptide (P1NP) and C-terminal telopeptide of type I collagen (CTX) are recommended reference bone turnover markers and could provide dynamic information beyond static bone mineral density measurements (Bhattoa et al., 2025). Future studies should measure these markers before and after initiation of ticagrelor, clopidogrel, or prasugrel to determine whether P2Y_12_ inhibition shifts the balance between bone formation and resorption. Serial sampling may also help identify patients with impaired fracture repair, as abnormal early changes in CTX or tartrate-resistant acid phosphatase 5b have been associated with delayed healing (Moghaddam et al., 2011). Emerging biomarkers such as collagen X degradation products may further capture the endochondral ossification phase of fracture healing and reveal drug-specific effects on callus maturation (Working et al., 2025). Standardized collection protocols, assay harmonization, and antiplatelet-specific reference change values will be needed before biomarker-guided treatment decisions can be clinically implemented (Brown et al., 2022).

### 7.5 Long-term vision

The long-term research goal is to determine whether antiplatelet agents have reproducible and clinically meaningful skeletal effects across relevant populations and treatment settings. This will require prospective comparative cohorts and, where genuine cardiovascular equipoise exists, appropriately designed trials with prespecified skeletal, ischemic, and bleeding outcomes. Studies should distinguish agent-specific effects from treatment duration, baseline osteoporosis, cardiovascular comorbidity, concomitant medications, and fracture-related factors, and should evaluate systemic therapy separately from experimental local delivery. Until such evidence becomes available, cardiovascular indications and ischemic and bleeding risks should determine antiplatelet therapy. Osteoporosis and fracture risk should be managed through established bone-health pathways rather than agent-specific antiplatelet selection or switching.

## 8 Conclusion

Antiplatelet agents may affect skeletal biology through platelet-dependent and purinergic pathways, but the evidence remains heterogeneous and predominantly preclinical. Aspirin and clopidogrel show context-dependent or conflicting skeletal findings, whereas evidence for prasugrel and other agents remains limited. Selected *in vitro* and animal studies report ticagrelor-related osteogenic signals consistent with ENT1–adenosine signaling. In one retrospective cohort, ticagrelor-based dual antiplatelet therapy was associated with lower fracture risk than clopidogrel-based therapy, but residual confounding and the absence of BMD, bone-turnover, and healing assessments limit interpretation. No prospective study has established a skeletal advantage of ticagrelor or any other antiplatelet agent. Current evidence does not support antiplatelet selection or switching on skeletal grounds. Treatment should remain guided by cardiovascular indications, ischemic protection, and bleeding risk, pending prospective human studies.
